# Supplementary material for: Allosteric regulation and crystallographic fragment screening of SARS-CoV-2 NSP15 endoribonuclease
Source: Nucleic Acids Res. 2023 Apr 28;51(10):5255–70. doi: 10.1093/nar/gkad314 (PMC10250223; doi:10.1093/nar/gkad314)
Supplement: gkad314_Supplemental_Files [file gkad314_supplemental_files.zip › Supplementary datasets.zip!/224/Refine_9/01-BUSTER/Cycle-2/shell.01/R_Rfree.html]

# Rxpct factors in resolution bins at cycle 0


|  |  |  |  |  |  |  |  |  |
| --- | --- | --- | --- | --- | --- | --- | --- | --- |
|  | **<d> (Å)**  Rxpct\_Work | Rxpct\_Free | Rxpct\_All | |  |  |  |  |
|  | 15.0574 | 0.3594 | 0.4838 | 0.3637 |  |
|  | 8.6934 | 0.2131 | 0.2586 | 0.2157 |  |
|  | 6.7339 | 0.2082 | 0.2304 | 0.2095 |  |
|  | 5.6912 | 0.1854 | 0.2499 | 0.1875 |  |
|  | 5.0191 | 0.1411 | 0.2002 | 0.1447 |  |
|  | 4.5400 | 0.1249 | 0.1597 | 0.1267 |  |
|  | 4.1762 | 0.1351 | 0.1783 | 0.1375 |  |
|  | 3.8878 | 0.1476 | 0.1974 | 0.1498 |  |
|  | 3.6520 | 0.1599 | 0.2077 | 0.1622 |  |
|  | 3.4544 | 0.1579 | 0.2107 | 0.1610 |  |
|  | 3.2858 | 0.1669 | 0.2113 | 0.1695 |  |
|  | 3.1397 | 0.1742 | 0.2348 | 0.1769 |  |
|  | 3.0115 | 0.1867 | 0.2190 | 0.1881 |  |
|  | 2.8978 | 0.2029 | 0.2101 | 0.2032 |  |
|  | 2.7961 | 0.2063 | 0.2302 | 0.2074 |  |
|  | 2.7044 | 0.2178 | 0.2388 | 0.2189 |  |
|  | 2.6212 | 0.2217 | 0.2586 | 0.2239 |  |
|  | 2.5452 | 0.2387 | 0.2649 | 0.2402 |  |
|  | 2.4754 | 0.2618 | 0.3227 | 0.2654 |  |
|  | 2.4111 | 0.2786 | 0.2672 | 0.2781 |  |
|  | **Overall** | 0.1816 | 0.2231 | 0.1837 |  |

# Rxpct factors in resolution bins at cycle 1


|  |  |  |  |  |  |  |  |  |
| --- | --- | --- | --- | --- | --- | --- | --- | --- |
|  | **<d> (Å)**  Rxpct\_Work | Rxpct\_Free | Rxpct\_All | |  |  |  |  |
|  | 15.0574 | 0.3593 | 0.4831 | 0.3636 |  |
|  | 8.6934 | 0.2136 | 0.2583 | 0.2162 |  |
|  | 6.7339 | 0.2078 | 0.2278 | 0.2090 |  |
|  | 5.6912 | 0.1866 | 0.2510 | 0.1887 |  |
|  | 5.0191 | 0.1423 | 0.1959 | 0.1455 |  |
|  | 4.5400 | 0.1271 | 0.1591 | 0.1288 |  |
|  | 4.1762 | 0.1374 | 0.1761 | 0.1396 |  |
|  | 3.8878 | 0.1504 | 0.1991 | 0.1525 |  |
|  | 3.6520 | 0.1623 | 0.2071 | 0.1644 |  |
|  | 3.4544 | 0.1603 | 0.2129 | 0.1634 |  |
|  | 3.2858 | 0.1693 | 0.2114 | 0.1717 |  |
|  | 3.1397 | 0.1763 | 0.2335 | 0.1789 |  |
|  | 3.0115 | 0.1885 | 0.2170 | 0.1897 |  |
|  | 2.8978 | 0.2032 | 0.2083 | 0.2035 |  |
|  | 2.7961 | 0.2061 | 0.2315 | 0.2073 |  |
|  | 2.7044 | 0.2187 | 0.2400 | 0.2198 |  |
|  | 2.6212 | 0.2225 | 0.2559 | 0.2244 |  |
|  | 2.5452 | 0.2382 | 0.2601 | 0.2394 |  |
|  | 2.4754 | 0.2578 | 0.3171 | 0.2612 |  |
|  | 2.4111 | 0.2774 | 0.2664 | 0.2769 |  |
|  | **Overall** | 0.1830 | 0.2221 | 0.1850 |  |

# Rxpct factors in resolution bins at cycle 2


|  |  |  |  |  |  |  |  |  |
| --- | --- | --- | --- | --- | --- | --- | --- | --- |
|  | **<d> (Å)**  Rxpct\_Work | Rxpct\_Free | Rxpct\_All | |  |  |  |  |
|  | 15.0574 | 0.3593 | 0.4828 | 0.3636 |  |
|  | 8.6934 | 0.2141 | 0.2579 | 0.2166 |  |
|  | 6.7339 | 0.2080 | 0.2263 | 0.2090 |  |
|  | 5.6912 | 0.1880 | 0.2509 | 0.1900 |  |
|  | 5.0191 | 0.1436 | 0.1936 | 0.1466 |  |
|  | 4.5400 | 0.1288 | 0.1585 | 0.1303 |  |
|  | 4.1762 | 0.1391 | 0.1751 | 0.1411 |  |
|  | 3.8878 | 0.1521 | 0.1996 | 0.1542 |  |
|  | 3.6520 | 0.1633 | 0.2064 | 0.1654 |  |
|  | 3.4544 | 0.1618 | 0.2143 | 0.1649 |  |
|  | 3.2858 | 0.1707 | 0.2113 | 0.1730 |  |
|  | 3.1397 | 0.1771 | 0.2329 | 0.1796 |  |
|  | 3.0115 | 0.1889 | 0.2166 | 0.1901 |  |
|  | 2.8978 | 0.2032 | 0.2079 | 0.2034 |  |
|  | 2.7961 | 0.2056 | 0.2308 | 0.2068 |  |
|  | 2.7044 | 0.2181 | 0.2390 | 0.2191 |  |
|  | 2.6212 | 0.2221 | 0.2551 | 0.2241 |  |
|  | 2.5452 | 0.2375 | 0.2594 | 0.2388 |  |
|  | 2.4754 | 0.2556 | 0.3168 | 0.2591 |  |
|  | 2.4111 | 0.2766 | 0.2663 | 0.2761 |  |
|  | **Overall** | 0.1838 | 0.2216 | 0.1858 |  |

# Rxpct factors in resolution bins at cycle 3


|  |  |  |  |  |  |  |  |  |
| --- | --- | --- | --- | --- | --- | --- | --- | --- |
|  | **<d> (Å)**  Rxpct\_Work | Rxpct\_Free | Rxpct\_All | |  |  |  |  |
|  | 15.0574 | 0.3604 | 0.4959 | 0.3651 |  |
|  | 8.6934 | 0.2402 | 0.2648 | 0.2417 |  |
|  | 6.7339 | 0.2444 | 0.2670 | 0.2457 |  |
|  | 5.6912 | 0.2308 | 0.2948 | 0.2329 |  |
|  | 5.0191 | 0.1873 | 0.2356 | 0.1902 |  |
|  | 4.5400 | 0.1730 | 0.1925 | 0.1740 |  |
|  | 4.1762 | 0.1788 | 0.1885 | 0.1794 |  |
|  | 3.8878 | 0.1830 | 0.2312 | 0.1852 |  |
|  | 3.6520 | 0.1880 | 0.2325 | 0.1901 |  |
|  | 3.4544 | 0.1842 | 0.2272 | 0.1868 |  |
|  | 3.2858 | 0.1924 | 0.2299 | 0.1946 |  |
|  | 3.1397 | 0.1935 | 0.2185 | 0.1946 |  |
|  | 3.0115 | 0.1921 | 0.2137 | 0.1930 |  |
|  | 2.8978 | 0.2000 | 0.1961 | 0.1998 |  |
|  | 2.7961 | 0.2002 | 0.2273 | 0.2015 |  |
|  | 2.7044 | 0.2064 | 0.2298 | 0.2076 |  |
|  | 2.6212 | 0.2067 | 0.2416 | 0.2087 |  |
|  | 2.5452 | 0.2186 | 0.2397 | 0.2198 |  |
|  | 2.4754 | 0.2239 | 0.2945 | 0.2280 |  |
|  | 2.4111 | 0.2424 | 0.2599 | 0.2431 |  |
|  | **Overall** | 0.2052 | 0.2370 | 0.2068 |  |
